# Sampling and analysis methods for detecting and monitoring airborne plant pathogens and fungal diversity

**DOI:** 10.1099/mic.0.001775

**Published:** 2026-09-29

**Authors:** Jonathan S. West, Gail G.M. Canning, Rohan B.E. Kimber

**Affiliations:** 1 Rothamsted Research, Harpenden, AL5 2JQ, UK; 2 South Australian Research and Development Institute (SARDI), Adelaide, South Australia, Australia

**Keywords:** bioaerosol, biodiversity, eDNA, plant disease, spore dispersal, spore traps, Aerobiology

## Abstract

Many important plant diseases are caused by airborne spores. This review explains the aerobiology of plant pathogens, sampling methods for collection of airborne spores, merits of different post-capture diagnostic methods (microscopy, immunology and DNA-based diagnostics) and non-capture (optical or imaging) methods to classify airborne particles. Different sampling, downstream processing and diagnostic methods can affect the efficiency of detection of certain species due to differences in traits such as spore size, adhesive surfaces, electrostatic charge, hydrophobicity, physical toughness and copy number of target DNA sequences. These considerations and an understanding of dispersal must be understood by all engaged in practical or academic measurement of bioaerosols, whether for plant pathology, fungal ecology or to measure biodiversity. In addition to surveillance of changes in pathogen populations, for example, for antimicrobial resistance or pathotype, the review describes how air sampling can provide accurate and cost-effective forecasting of imminent plant disease. The scale of spore dispersal processes and impacts on sampling strategies (e.g. spatial density of spore traps and sampling height above ground) for broad-acre or small-scale but high-value crops is also discussed. The fundamental concept behind these processes is the aerobiology pathway, which describes the production, release, dispersal, deposition and resulting effect, which, for air-dispersed plant pathogens, is plant disease. In addition to the seasonality to spore production for many fungi, each component of the aerobiology pathway is affected by the weather, which causes different types of spores to be released under different meteorological conditions or at different times of day and to disperse in different ways. Dispersal processes of fungal spores in air have been modelled and apply irrespective of pathogens or non-pathogenic fungi, or to spores of individuals that are insensitive to fungicides or not. Often, we do not know the location of spore sources or their relative source strength, which makes sampling strategies difficult to optimize initially, and the best strategy will vary from pathogen to pathogen and according to the rationale for monitoring and consequences of ‘missing’ a spore event.

**Impact Statement**

In over 70 years of research using spore traps (rotorod, cyclone and the Hirst spore trap) to study associations between airborne spores, the weather and crop diseases, many new researchers, mostly using DNA-based methods, some using image analysis, will benefit from a review summarizing key considerations in sampling, analysis and interpretation of results. This timely review summarizes those fundamental considerations of the aerobiology of plant pathogens, which is important for policy-makers, those monitoring bioaerosols for practical plant disease warnings, to monitor biodiversity, or to assess specific populations for changes in traits such as antimicrobial resistance. The article describes the aerobiology pathway, including the scale of spore dispersal processes, active and passive spore-release mechanisms and their impacts on sampling strategies (e.g. time of day, spatial density of spore traps and sampling height above ground) for pathogens of broad-acre or small-scale crops. It also reviews the current state of art in sampling methods for collecting airborne spores, merits of different post-capture diagnostic methods (microscopy, immunology and DNA-based diagnostics) and automation of spore detection, including non-capture (optical or imaging) methods. Spore dispersal processes are the same for pathogens or non-pathogens, those insensitive to fungicides or not, but our missing knowledge is often the location and relative strength of spore sources.

## Data Summary

The authors confirm that all supporting data, code and protocols have been provided within the article or through supplementary data files. No unpublished datasets were used in this review.

## Introduction

It was already established by the early nineteenth century that pollen of many plants, spores of lower plants such as mosses and ferns, most fungi, many protists such as oomycetes and bacteria were dispersed in air along with fragments of plants and animals [1]. Methods used for monitoring airborne biological particles were developed by Pasteur, Maddox, Cunningham, Schoenauer and Miquel in the 1800s [2]. More recently, particularly since the 1950s, methods for continuous outdoor sampling with volumetric spore traps were developed to monitor plant pathogens and allergenic pollens [3–5] and recently to assess biodiversity and antimicrobial resistance. Developments to monitor airborne plant pathogens have been important to improve strategies to control plant diseases, which reduce yield, quality and economic return on crops with losses estimated at 16–20% globally despite great efforts by farmers to control diseases [6, 7]. In addition to crops, plants in other terrestrial ecosystems, such as forests, urban and wild landscapes, can suffer from diseases caused by a range of pathogens, including viruses, bacteria, fungi, oomycetes, nematodes and, in a few cases, algae [8, 9]. Many of these pathogens are dispersed by airborne spores or inadvertently dispersed in air as fragments or dried particles mixed with soil dust, particularly during harvest or other operations, or as a wet aerosol created by heavy rain events combined with wind. In the case of some viruses and some bacteria, they can also be airborne either individually or vectored by other airborne particles such as pollen or spores that they have either infected or are carried on. Airborne dispersal distances vary greatly due to a combination of atmospheric effects, such as wind speed, presence of a filtering crop canopy and the ability of pathogens to withstand UV light, desiccation and extremes of temperature, which results in some pathogens being able to disperse over oceans or continents, while others may typically disperse relatively short distances as viable particles [10–12]. This article aims to review aspects of short- and long-range airborne dispersal of fungal plant pathogens and the processes affecting their dispersal and implications for current methods used for sampling and downstream diagnostic methods to identify and quantify them. The review does not cover plant diseases vectored by insects or spread by humans on imported plants, seeds or machinery.

Crop diseases are usually controlled using an approach known as integrated pest management [13], which comprises the manipulation of key factors to reduce diseases such as crop rotation to separate the same host plants in space and time and therefore reduce a new crop’s exposure to airborne inoculum (the propagules of a pathogen that cause disease), adoption of resistant varieties, use of varietal mixtures and intercropping, or to apply crop protection products (biocontrol or chemical pesticides), which can even have synergistic effects [14]. Critical to these practices is effective and accurate surveillance and diagnosis of the causal agents, which directs the correct interventions by farmers to minimize disease spread or impact on yield. Sampling air for plant pathogens is an important surveillance and monitoring method to forecast imminent disease risk. This can be based on detecting airborne inoculum directly, which facilitates alerts to ensure good timing of crop protection products before infection or at early stages of infection, or spore trap data can be used with climatic data to develop weather-based forecasts [15–19]. In addition, air sampling can be an efficient way to monitor changes in pathogen populations for genetic traits, e.g. conferring fungicide resistance [20, 21] or ability to infect specific crop varieties (known as pathotypes) [22]. Therefore, innovations for detecting and monitoring airborne plant pathogens are an important part of integrated pest management and contribute to food security by helping to reduce crop diseases. An understanding of spore release and dispersal processes is important to optimize sampling strategies for both pathogen surveillance and assessment of fungal diversity to assess changes in pathogen populations or, more broadly, as a measure of biodiversity.

### Plant pathogens and the aerobiology pathway

The aerobiology pathway comprises processes causing spores to be released from their source, dispersed downwind and then deposited to cause an impact such as plant disease [23]. It is important not to consider the air itself as a source of biological particles, but instead, the air should always be considered as one of the pathways delivering spores that were released from the true source where the organisms grew and reproduced. This is important from the perspective of control and mitigation. Each component of the aerobiological pathway is affected by environmental conditions and is difficult to measure alone. Spore dispersion is a spatiotemporal process that operates across numerous scales in space and time with success dependent on the factors that influence liberation, escape, transport, survival and deposition of propagules [24].

Three main methods of spore release are passive, active and rain-splashed. Passive release is where a gust of wind penetrates the layer of still air (laminar boundary layer) close to surfaces with sufficient force to detach the outermost spores in a pustule, causing them to become airborne. Spores such as conidia of powdery mildews, *Cladosporium* and *Botrytis* and urediniospores of rusts (e.g. *Puccinia* spp.) are normally released this way, having been produced during humid night-time conditions, and are typically released and reach high concentrations in the air during the middle of the day when the air is driest and wind speeds are greatest [1]. In contrast, active release can occur by a number of different mechanisms, such as explosive puffs of spores caused by a dehumidification event (typically caused by a gust of relatively dry wind into a humid plant canopy) or moisture absorption response, such as the common mechanism in ascomycetes where spores can be shot outwards by turgor pressure within a fruiting body after wetting by rain or dew [25]. As a result, many actively released spores are also associated with certain weather events within their season of production, e.g. Fig. 1 showing an association of *Sclerotinia sclerotiorum* spores with dry weather after rain events, as the fruiting bodies need moisture to grow and mature but release their spores when humid air is removed by a gust of drier air. In the case of *Sclerotinia*, like many fungi, continuously dry weather eventually prevents spore production because the fruiting bodies dry up or stop being produced. Additionally, for many actively released spores, there is a diurnal periodicity with peak release occurring at different times of day for different groups with spore peaks ranging from early morning, mid-day, post-dusk or night-time [26]. Although daily periodicity, seasonal occurrence, weather events associated with spore release and associations of airborne spores with crop diseases were reported for many microbes since the 1950s, new diagnostic methods, particularly metagenomic sequencing, are enabling many more microbes to be characterized in terms of spore release pattern and distribution (e.g. [27, 28]).

**Fig. 1.**
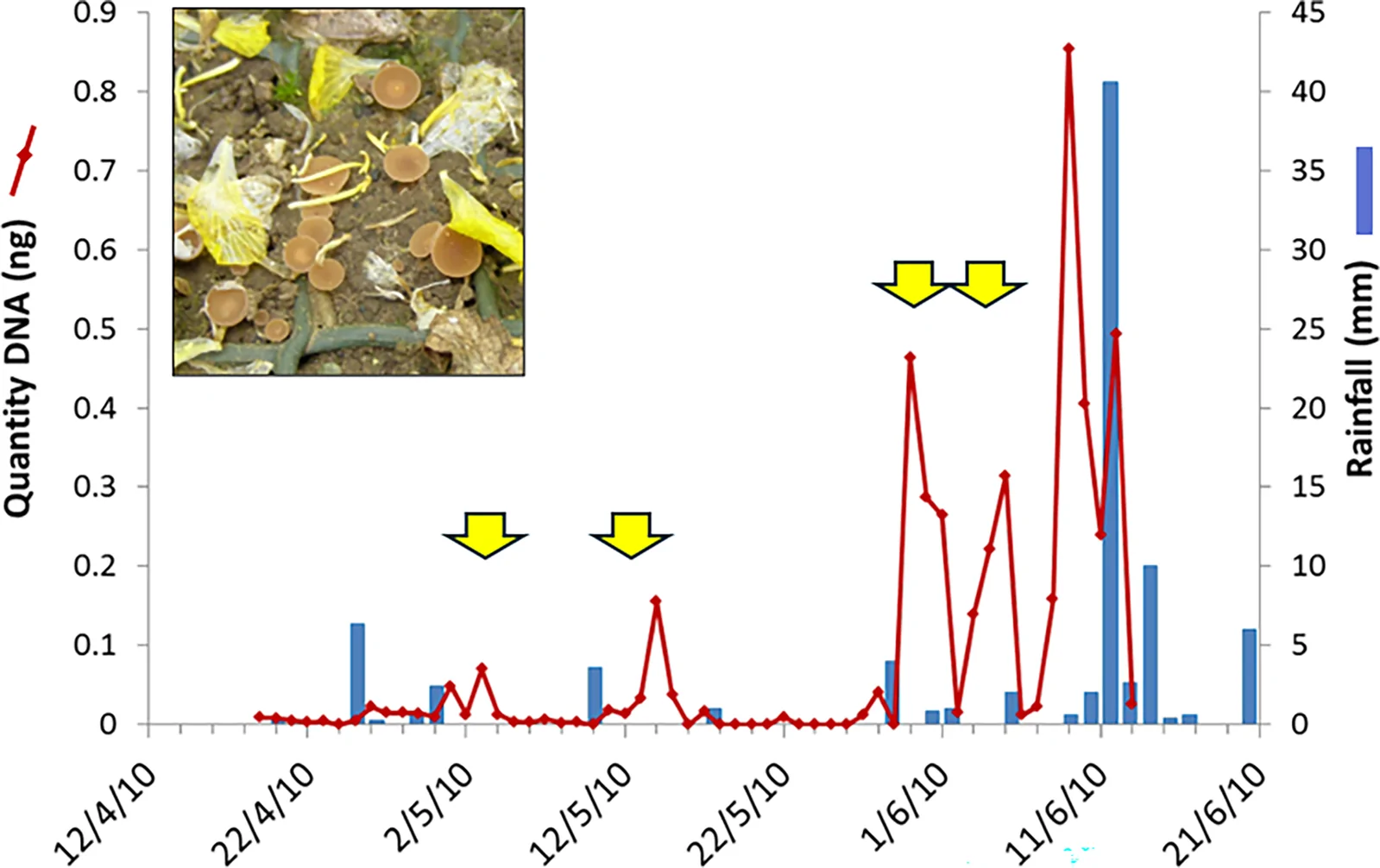
Pattern of *S. sclerotiorum* DNA (red line, indicating airborne spores) from daily Burkard spore trap samples collected at ADAS High Mowthorpe, UK, in 2010 and association of the airborne spores with dry weather (yellow arrows) after rain events (blue bars). The inset photo shows the fruiting bodies (apothecia) which actively release spores in a visible puff when relatively dry air blows over them, improving dispersal. Note that no spores were produced in the dry period before 24 April because the apothecia had not yet developed, nor after 13 June because all the fruiting bodies had died out. Adapted from [118].

A form of dry dispersal can also occur when raindrops jostle leaves at the start of rain events, causing mechanical release of dry spores into the air, known as rain puff. This also occurs in glasshouses and polytunnels when drops of condensation fall onto leaves. The third main type of spore release is rain splash. Rain is more commonly associated with both splash dispersal of spores that are mixed into raindrops and the creation of a wet aerosol, particularly when rain is combined with wind. The former type, known as ballistic splash, disperses spores typically <2 m, but the latter, wet aerosol with spores mixed into fine droplets, can disperse spores tens and even hundreds of metres [29, 30]. In theory, a fine spray of wet aerosolized spores can evaporate after a rain event to leave spores suspended in air as a dry aerosol.

Having been released, spores disperse downwind and dilute in the air as they travel due to a combination of deposition onto surfaces and dilution upwards and sideways into the air as they travel. Movements of spores downwind in air are a complex combination of air mass movement (wind), turbulence (leading to eddy diffusion), thermals and gravity. Spore size has a relatively small effect on dispersal of fungal spores (ranging in aerodynamic diameter from 3 to 30 µm) compared to effects of air mass movement, and spore shape has an even smaller effect, but generally, smaller spores are more easily dispersed in gentle air currents [31]. The resulting spore dispersal gradients are typically negative exponential or inverse power functions, which both have a common feature of relatively high concentrations close to the source but declining with distance to low concentrations [32, 33]. The exact shape of the spore gradient with distance depends on the weather at the time of spore liberation, the density of crop canopy and the size of the source. Of these, laminar wind causes negative exponential gradients when plume depletion is predominantly by deposition in the crop canopy, but inverse power functions occur in more turbulent conditions [32]. The size of source has an impact due to lateral diffusion of the spore plume downwind, where small point sources have a steeper decline due to eddy diffusion sideways as the spore plume disperses, compared to a shallower dispersal gradient from area or line sources which, over the central part of the plume, do not lose spores outwards by eddy diffusion as any lateral movements are compensated by spores diffusing inwards from spores released further to the side [31]. On larger scales, there may also be an effect of rows of trees and local topography, influencing wind direction [24, 34]. In addition to the physical dilution of spores in air with distance from the source, there is also a decline in their viability depending on their ability to survive under varying relative humidity, temperature and UV light. So, in terms of viable particles, it is necessary to superimpose a survival function (or kernel) with time onto the physical particle dispersal curve, which means a disease gradient downwind of a source can be steeper than the physical spore gradient might suggest due to mortality of some spores as they travel downwind [35].

Deposition of spores can occur by impaction onto surfaces such as plants, passive settling in still conditions and, in some instances, spores acting as condensation or ice nuclei. In the latter case, the spore acting as the nucleus ends up falling to the ground in snow or more usually in rain [36]. Falling rain can also impact free airborne spores and cause them to be washed or ‘scrubbed’ out of the air by the rain event [25]. Given the correct conditions for infection, and assuming the spore remained viable and had landed on a susceptible host plant, the aerobiological pathway for a plant pathogen is completed by infection of the host, leading to new sporulation. It is difficult to measure individual components of the aerobiological pathway because spore samplers capture the combined product of spore production (source strength), spore release and dispersal from the source to the sampling position. The use of eddy covariance measurement has not been used greatly in plant pathology or fungal ecology to estimate the relative source strength of spores being dispersed from a source in the same way as used to quantify the outdoor production of greenhouse gases [37, 38].

### Sampling airborne plant pathogens and *in situ* analysis

Air sampling devices can be divided broadly into two types – passive and volumetric (or active). Passive and volumetric samplers are discussed in other reviews [39, 40], but to summarize, key points to consider are as follows: the volume of air sampled per minute; capture bias – i.e. the cut-off aerodynamic diameter below which <50% of particles of that size will be captured, which differs from device to device; ease of downstream post-capture processing and cost of the trap and its operation. The advantage of volumetric traps is that the volume of air sampled is known, and so a fundamental aspect of aerobiology can be met – that the concentration of spores in air can be calculated as a number of spores per unit volume of air, e.g. 50 spores per m^3^. That calculation is not possible with passive traps. Passive traps rely on spores either impacting or settling onto a collection surface. This could be a sticky mesh, rod or glass slide, protected from rain in a piece of pipe or under a rain shield. Air flows around obstacles, but the momentum of particles in the air causes them to continue onwards for microscopically small distances to impact onto the collection surface or to settle onto surfaces in still conditions. With broad obstacles, the air flow changes direction earlier than for narrow obstacles, and so narrow or thin surfaces are better at collecting spores by impaction than broad surfaces because particles, particularly smaller particles, are more likely to move with the air and miss the collection surface if it is broad. The simplicity and low cost of passive traps mean they are cheap and can allow comparative studies to look for spatial comparisons of airborne spores either with other passive traps or additional volumetric traps. Some semi-passive traps are starting to be used and may rely on an internal low-cost solar-powered fan to augment what would be captured passively, e.g. the Spornado system [41], which captures spores on a fine mesh.

A common principle of volumetric spore sampling is impaction onto a sticky surface. This is used in the Hirst or Burkard spore trap, rotorod trap, Microtitre Immuno Spore Trap (MTIST), cascade impactor or Andersen sampler [39, 40]. Other main forms of volumetric trap are electrostatic traps, wet and dry cyclones, liquid impingers and filters. Wet cyclones and especially liquid impingers are particularly good for collecting small particles as the air makes intimate contact with liquid or wet surfaces and collects particles into the liquid or wetness film as a result. A drawback is that the liquid needs to be topped-up to account for evaporation if the sampler is being used for a long period. Another device useful for the collection of very small particles and gentle collection into liquid to maintain viability is the water condensation particle growth technique, e.g. the Series 300 BioSpot Bioaerosol Sampler, which collects particles as small as 5 nm–10 µm and has been used to sample airborne viruses, bacteria and yeasts for clinical and defence purposes but has not been used routinely to sample plant pathogens [42]. Filters can also collect very small particles, but often the airflow sampled is low due to the restriction caused by the filter, unless the filter surface area is very large, which then causes issues with recovery of the sample from the filter surface (discussed later). An alternative is to use a filter that can be dissolved in a suitable solvent. Parker *et al*. [42] used a gelatine filter, which can be dissolved to release particles, but these filters can dry up during operation in dry airflows or dissolve in very humid air.

Many recently developed types of spore trap have tried to reduce post-capture processing, mainly by capturing samples directly into tubes, which is an advantage of the cyclone or wet cyclone and MTIST. The multivial miniature cyclone was developed to capture spores into 1.5 ml sample tubes arranged on a carousel with a simple timer to enable a new tube to be used each day for over a week [43]. The MTIST captures spores directly into wells of microtitre plates [44]. In all cases, it is necessary to know the collection efficiency of a device for the spore size under investigation so that the concentration per cubic metre of air can be estimated based on the air volume sampled and the collection efficiency for the particle of interest. There is some confusion over collection efficiencies of rotorod spore traps, particularly because there are many different designs, which each have different collection efficiencies. Those with relatively narrow collection surface that are moved quickly through the air (a function of speed of rotation and circumference of the rotor) have the best collection efficiencies. For example, those used by the authors collect onto rods 1.6 mm wide by 34 mm long and rotate at 1,700 r.p.m. with an external diameter of 104 mm and therefore sample 60.4 l min^−1^ (for two arms) and seem to have very similar collection efficiency to the Hirst type spore trap (with 2×14 mm inlet) for particles ranging from 3 to 30 µm (unpublished observation). Whereas collection efficiencies often quoted for rotorods give an EC_50_ value (diameter below which less than 50% of particles are collected) of 20 µm.

Another development has been to manage spore trap operation with electronic control systems, particularly for use in remote locations where mains power is not available, to preserve battery life and, in the case of rotorod spore traps, to avoid sample arms becoming overloaded with dust during extended sampling periods. These systems can be programmed to sample cyclically, e.g. 1 min on and 9 min off during the sampling period [45] to mitigate this. However, certain spores can be released at different times of the day to others, so the use of timers can cause different collections, e.g. Reich *et al*. [45] found that different airborne fungal communities were collected at the same locations in Canada between a Burkard miniature cyclone and Aerobiology Research Laboratories GRIPST-2009 rotating-arm (rotorod) spore traps, most likely due to the cyclone running for 24 h per day while the rotorod only sampled for 8 h per day.

In the last decade, technology advances have allowed for greater automation, including continuous or same-day monitoring of airborne spores using a range of volumetric, smart (internet-linked) systems, many of which rely on some kind of optical or image-based classification or machine learning to identify airborne particles, in some cases, as particles pass by a detection sensor rather than being captured for analysis. Although the complexity of technology integrated within these devices inevitably increases their size and cost, their potential in terms of providing continuous or same-day information without needing a technician or daily reagents is very appealing. The Hund BAA500 [46] works by taking a holographic image of particles, which can be classified into broad types and possibly identified to individual genera and even species, e.g. *Alternaria* species [47]. Similarly, the Plair RapidE [48] builds on the earlier development of the Wide Issue Bioaerosol Sensor [49], and similar recent devices are called ‘Wideband Integrated Bioaerosol Sensors’, to use fluorescence of individual particles in a narrow stream of air flowing through the device under UV and red laser excitation, along with the particle size and shape to diagnose the particle type [50]. Light scattering and fluorescence of particles are also used in the Swisens Poleno [51]. Trials are currently underway to evaluate these devices particularly as part of the SYLVA project [52], which has developed guidelines for culturing and creation of a reference set of spores, including a design for a simple home-made cyclone spore collector [53]. Generally, these optical systems have shown great promise for identifying many pollens, but considering that there are an estimated 2.2–3.8 million species of fungi [54], some of which produce more than one type of spore, they could be restricted to identifying broader ‘spore types’ which, with some context, such as proximity to a certain crop, might serve as useful information to farmers and agronomists in the future. Similarly, MALDI-TOF MS has been used to identify different microbes in environmental samples, including in air, but further work is required to assess its potential to identify plant pathogens within complex mixtures of airborne microbes [55].

A less costly system, also under evaluation for area-wide deployments within agricultural landscapes, is the Bioscout system [56], which integrates light microscopy, digital imaging and machine learning algorithms to autonomously classify particles impacted similarly to a Hirst spore trap at 10 l min^−1^ onto an adhesive tape each day. The results are communicated to subscribers via an online dashboard showing same-day information on concentrations of spores of interest at each site. The SporeCam^™^ from Scanit Technologies [57] also works by imaging particles impacting at 10 l min^−1^ via a fixed directional air intake and sending algorithm-based results to the cloud. Results so far are limited to one published study showing a mixed response for three grapevine pathogens tested – negatively correlating with rotorod spore traps for *Plasmopara viticola* but with positive correlations for *Botrytis cinerea* and *Erysiphe necator* [58]. Another imaging system called SporeSense [59] induces spores to germinate on a surface mimicking a leaf and can identify spores of *Puccinia striiformis* (yellow or stripe rust of wheat) based on the pattern of germ tubes formed by germinating spores. The potential advantage this has is that detected spores are shown to be viable, but further work is necessary to add detection of other key species. In many cases, detection of large numbers of a particular spore usually indicates that they arise from a local source, which also implies that at least some of the spores will be viable, which is why many DNA-based methods have worked well. However, in theory, there could be instances of spores being detected that come from a large release from a distant source where an indication of viability could be important. Sporangia of *Phytophthora infestans*, for example, may only survive 1 h in strong sunlight [33].

Fig. 2 shows how these contrasting systems have been developed since the 1950s, comprising devices for continuous imaging or spectroscopy methods, processing of spore trap samples for DNA-based diagnostic methods, including sequencing and in-field automated systems currently under development [60] and applications of immunological methods, which are also starting to be automated [61].

**Fig. 2.**
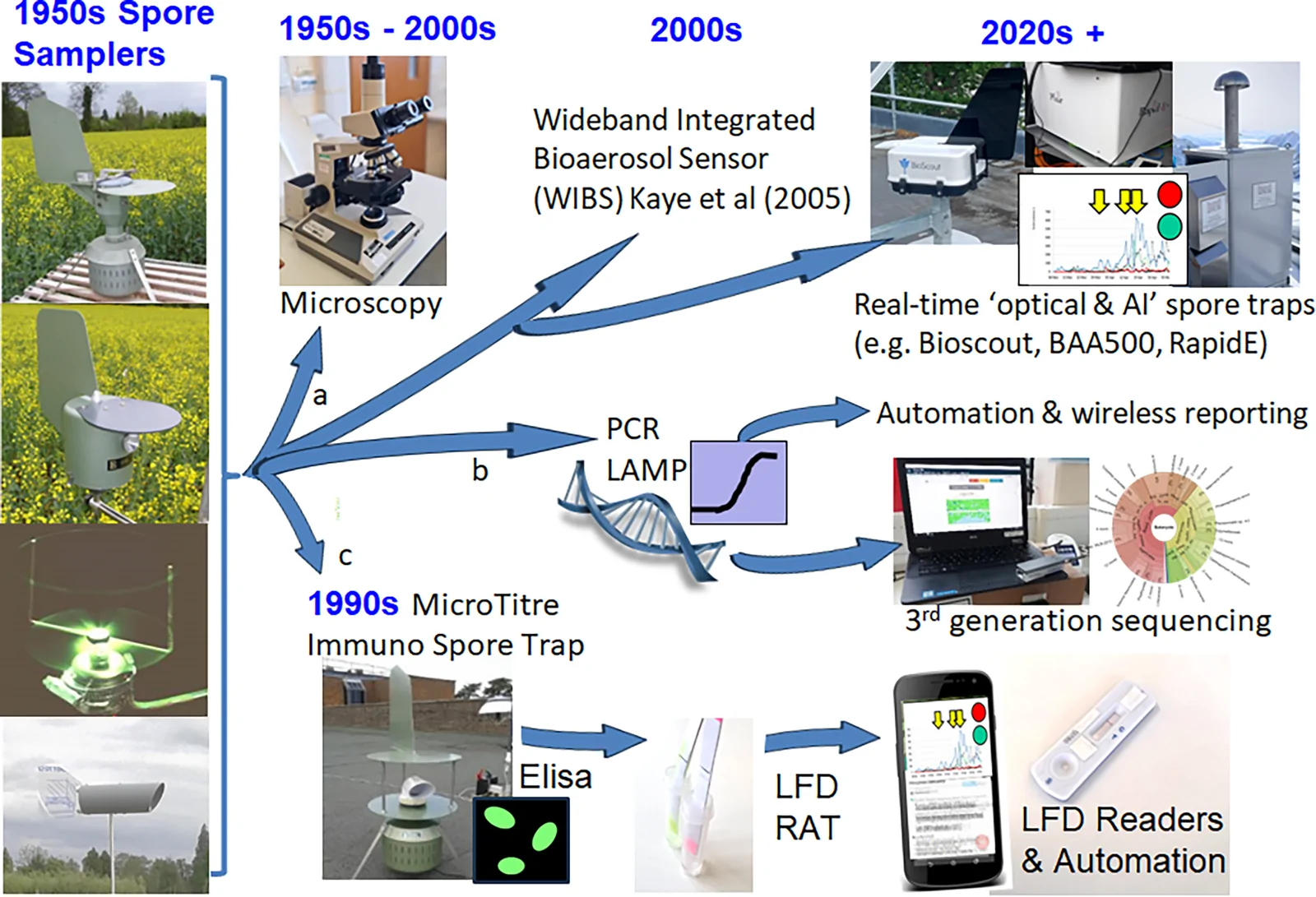
‘Evolution’ of different samplers and diagnostic methods used for monitoring airborne spores since the 1950s, broadly divided into (**a**) imaging or spectroscopy systems, (**b**) DNA-based methods and (**c**) immunological methods.

### Deployment of samplers

To optimize spore sampling, spore traps are often placed about 20% higher than the crop canopy, as this gives the best combination to capture both spores released from the crop canopy and those arriving from more distant sources [62]. However, for practical reasons, the air intake or sampling surface needs to be at least 50 cm above ground level even for a very short canopy such as a newly emerging crop, to reduce intake of soil particles and rain splashes. Trapping spores at two different heights can be used to infer whether the spores are coming from the crop canopy or blowing from an external source because spores arriving to a crop canopy of, say, 50 cm height from an external source are likely to be at similar concentrations at both 1 and 5 m, whereas spores released very locally will be at a much higher concentration at, say, 1 m compared to 5 m [63, 64]. Similarly, because air near the ground is heavily weighted to locally released spores, whereas air at, say, 10 m comprises more mixed air containing spores and other particles released over a larger ‘footprint’, sampling at 10 m has found approximately double the species richness of that at 1 m [65]. This could be good for low-cost surveillance of common pathogens but potentially is not ideal for the purpose of biodiversity studies if the ‘footprint’ of the sampler is too large. For example, an investigation using spore traps combined with DNA sequencing of samples collected in 2012–2013 using a sparse network of rooftop spore traps at Rothamsted (UK), Wageningen (NL) and Slagelse (DK) found that despite the traps being up to 900 km apart, remarkably similar compositions of fungal spores were in air, which varied only slightly between the three sites due to timing of weather events, despite large seasonal variations [65].

The area represented by a spore trap is complex to explain and varies with the weather, the height of the sampler above ground level and the type of vegetation surrounding the trap. A spore sampler can be imagined to be sampling a chain of small ‘boxes of air’ that are flowing from many miles or kilometres upwind, finally to go into the spore trap (Fig. 3). As the boxes of air move ever closer to the spore trap, spores already in a box may leave by continuing to diffuse upwards, sideways or downwards, while at the same time, new spores may diffuse into the box of air. So the final composition of air going into a spore trap near ground level will be a relatively small proportion of spores that have travelled a very long way (e.g. 3–300 km), a moderate number that have travelled an intermediate distance (e.g. 300 m–3 km) and many that have travelled a relatively short distance (1–300 m). In contrast, a spore trap on a rooftop at, say, 10 m height above ground level is sampling more mixed air, which will have a different composition of spores to that near the ground, in particular with fewer spores produced close to the trap and more that have come a relatively long way (Fig. 3).

**Fig. 3.**
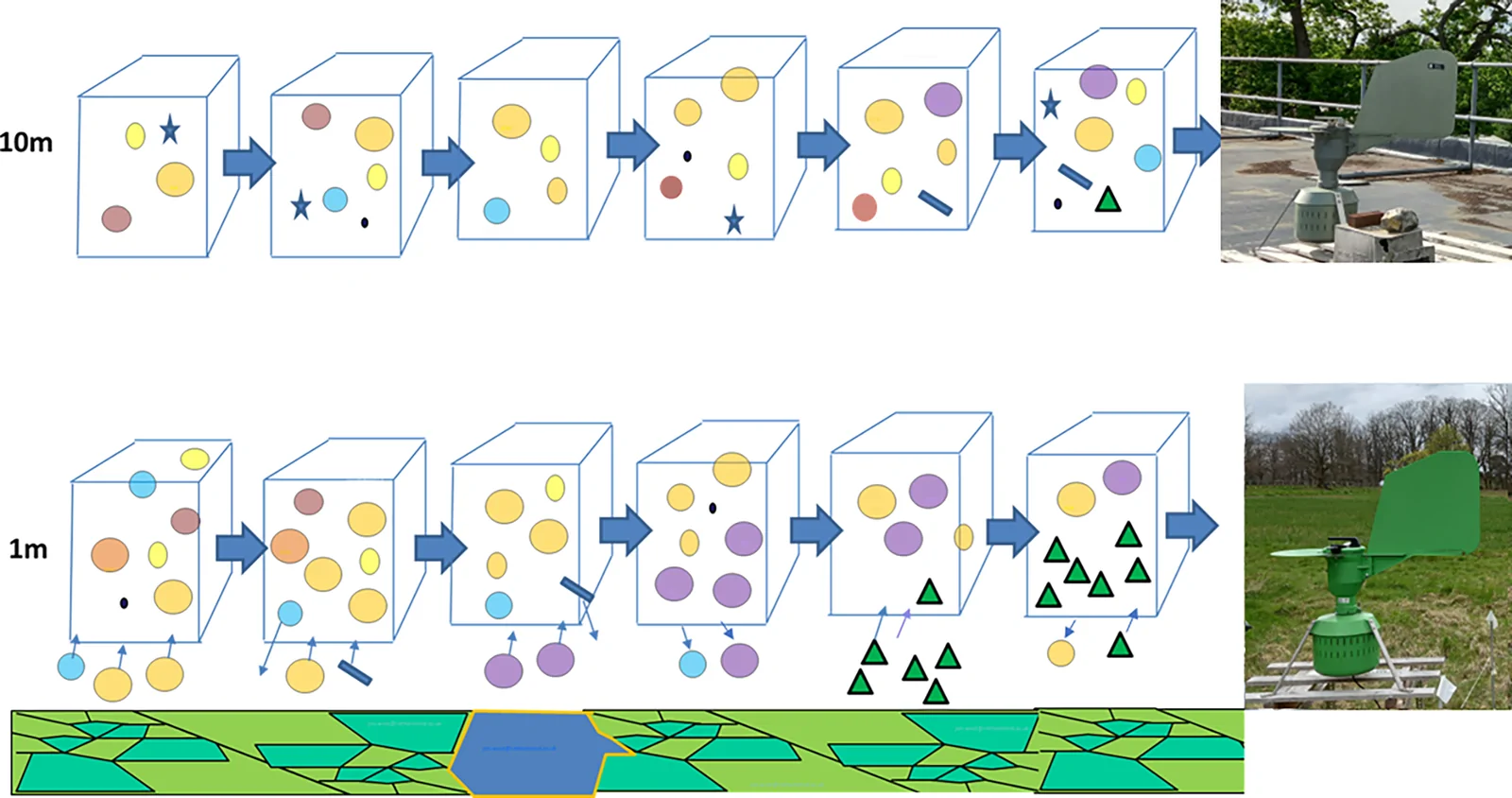
Depiction of the composition of biological particles in imaginary ‘boxes of air’ as they move by the wind towards a spore trap, where the air is sampled (note that the spores, heights and spore traps are not to scale). The fungal spore species richness is greater at 10 m than at 1 m, but the concentration of spores produced close to the sampling position is greater near to the ground.

Due to the complex airborne particulate dynamics shown in Fig. 3, a spore trap samples an area immediately upwind of it, but an elevated spore trap can be considered to have a larger ‘footprint’ in terms of the area it represents. In addition to the area represented by a spore trap, another consideration is the importance or value of the crop or crops the trap is being used for. For example, for deployment of spore traps to provide information on the timing of common pathogens of widely grown crops, e.g. *Zymoseptoria tritici* in a large wheat-production area, potentially one trap could reliably inform decisions on an entire region, where it will act as a ‘barometer’ or proxy, to indicate the presence of a regional ‘cloud’ of spores of a common pathogen. However, to provide reliable information on airborne pathogen risk for a rare, high-value crop, it would be necessary to have a spore trap within the crop and, if the value of the crop is high enough, even multiple samplers in the same field to ensure detection of a small, localized plume of spores arising from a small point source nearby or in the field itself (Fig. 4). Where there is sufficient density of sources within a landscape (e.g. Fig. 4a), an area effect occurs in which dilution downwind of an individual field-sized source causes the spore concentration to decline not to zero but to a regional background concentration that is substantially above zero (e.g. Fig. 4d). This decline to the regional background occurs typically within 300–2,000 m of the source [33, 66] but it can take a greater distance to decline to the regional background for particularly large sources. In contrast, where there is only one or a few small sources within a landscape (Fig. 4b, c), the regional background concentration is likely to be close to zero (Fig. 4e, f), which is why it is necessary to have a spore trap or traps within the field that is under surveillance. For example, a pattern similar to Fig. 4e, f was found for *Eutypa* and *Botryosphaeria* spores in a vineyard using rotorod spore traps with the best detection site on the southern edge of the vineyard or 50 m further south due to a predominantly northerly wind pattern [67].

**Fig. 4.**
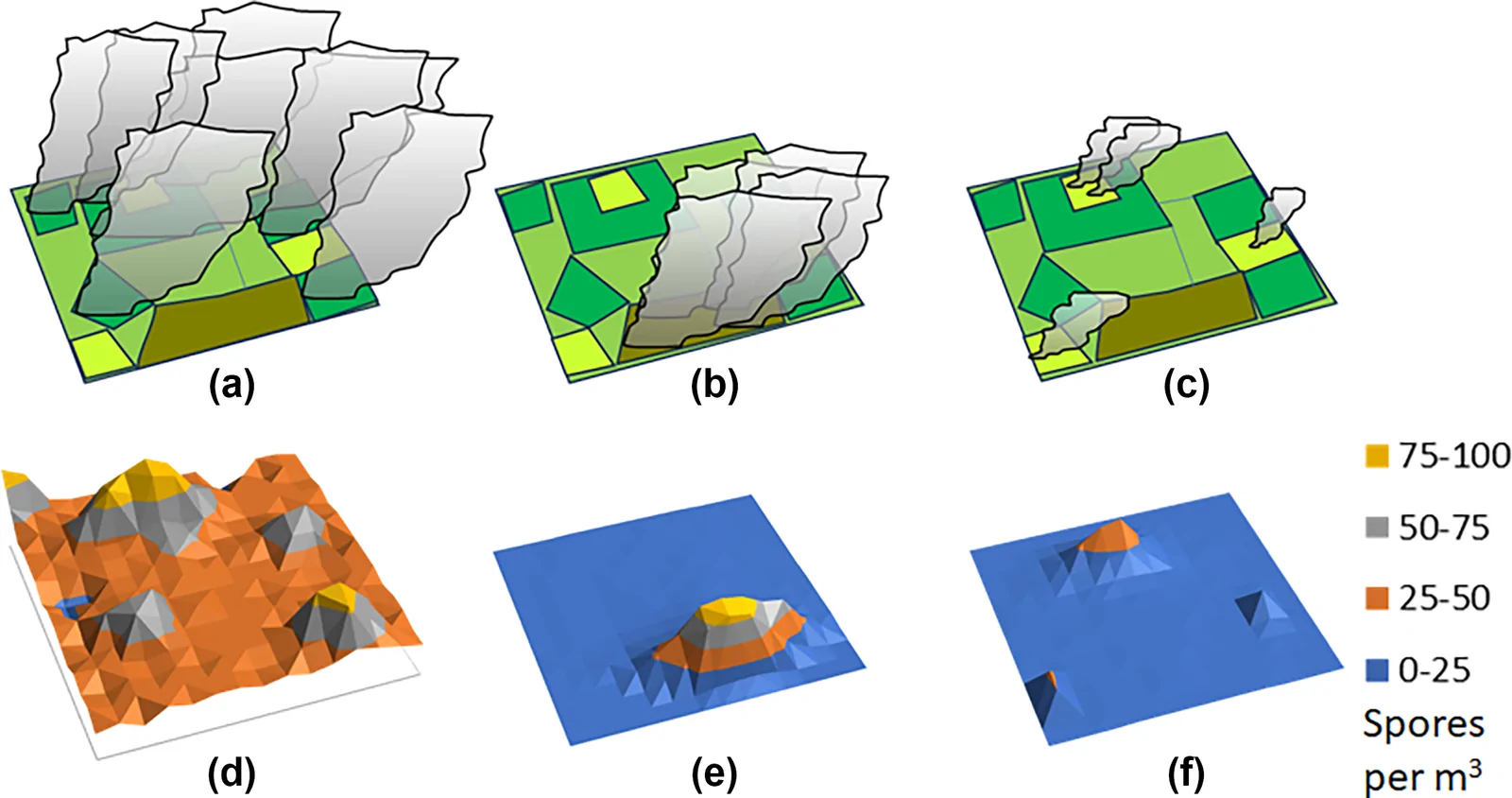
Simple visualizations of plumes of airborne spores of different species (**a–c**) being released from different sources in a landscape and corresponding contour plots simulating the respective spatial spore concentrations near to ground level, i.e. height indicates spore concentration near ground level (**d–f**). Species (**a**) is a common pathogen of a widely grown crop and could be detected by a single spore trap placed anywhere in the landscape, whereas species b and c would only be detected by a spore trap in each field where the spores were produced or immediately downwind of those sites because elsewhere, the respective spore concentrations of those species are close to zero.

As Aylor summarized, without prior knowledge of the distribution of spore sources in a crop or landscape, ‘the initial deployment of samplers is pretty much of an art and must be refined after the first data are obtained’ [33]. The need to understand spatial scales of inoculum dispersion processes was also emphasized for each different target species [40]. Therefore, the idea of using networks of static traps is appealing as they reinforce each other in terms of the information they provide, but the density of the network would depend not only on the spatial scale of dispersal processes for the pathogen in question but also on the value of the crop, the crop’s prevalence and financial impact of missing a spore event, which also depends on the window for effective control of the pathogen in question – for example, potato blight which has potential to devastate a crop very quickly if conditions are conducive would necessitate a zero risk strategy compared to a cereal crop exposed to a rust or powdery mildew, which are less devastating and more easily controlled. Ideally, when researching spore patterns of a new disease, spore traps should investigate airborne spore concentrations at different scales if resources permit, but otherwise, local farmers usually know where disease hotspots are, which can be due to microclimate, exposure to primary inoculum or both. Nevertheless, even quite sparse networks can be informative in terms of the seasonal timing of spore release in different climatic zones of a country; for example, 5 trap sites were used in the south of Belgium to monitor *Fusarium* species affecting wheat [68], 3 sites were used in Canada to assess fungal diversity [69], 12 sites were used in France to monitor tree pathogens using metabarcoding and qPCR [70], and 4 sites were used in central and east Poland to develop models predicting seasonal ascospore release by *Leptosphaeria maculans* [71]. However, the advantage of much denser networks, apart from not missing a small local spore event, is to allow the differentiation between detection of a large, distant source from one or more small local sources. Examples of such networks providing practical information to growers include the Quebec onion disease network, which has traps distributed on a 30 km grid scale [72], or the Brassica alert network, which has four spore trap sites in an area ~40×20 km region of southern Lincolnshire in the east of the UK, and the commercial service provided by Root Applied Sciences to vineyards in California [73].

In the case of biosecurity monitoring, i.e. detection of a rare exotic pathogen arriving in a new territory, spore traps have their limitations due to the relatively insignificant volume of air they sample [74]. Spore traps will always struggle to detect very rare spores but do seem to work well at detecting variations in the seasonal timing of common pathogens. The biosecurity aspect might be improved using high-volume spore traps and raising their sampling height and also by sampling rain, which often brings long-distance dispersed spores back to ground level [69]. For the purposes of monitoring allergenic pollen and fungal spores, many national networks were established, and most have tended to use rooftop locations at 10–30 m height due to this height representing the air spora of the region, buffered or smoothed from the effect of small, local sources and also with the practical advantage of mains power, less noise for neighbours and good site security [75].

Networks of static traps can be programmed to sample the air either continuously or at least for key periods of the day when the target spores are likely to be present, e.g. early morning to mid-afternoon for a passively released spore such as a rust or powdery mildew. Additionally, mobile traps can be used to provide more spatially specific information on spore sources, although due to some spores being released at certain times of day or after certain weather events, there is a risk that mobile traps could miss a spore event. Nevertheless, vehicle-mounted spore traps have shown great promise and were shown in one study to be superior to a network of static spore traps [76]. For example, Limpert *et al*. [77] used the Schwarzbach jet spore sampler mounted on a car, and the same device is still used routinely to monitor key pathogens in Europe to assess fungicide resistance and survey pathotype distribution [78]. The ‘Jet’ spore sampler is a type of virtual impactor, and a modification of that was developed to deposit spores into DNA extraction tubes loaded on a carousel which is controlled by a mobile phone app to switch tubes when the vehicle is driven into a different region of a planned route [79]. Spore traps can also be used on drones and remotely piloted aircraft [80–82], aircraft [10, 83] and balloons [84, 85].

### Post-capture processing and diagnostic methods

This section concentrates on practical approaches where a collected sample is processed for a diagnostic technique such as an immunological, aptamer or DNA-based method to be applied to an air-particulate sample, rather than automated spectroscopy or imaging, which was mentioned in the section on air samplers. Many volumetric spore traps developed in the 1950s were designed for microscopy to identify spores of plant pathogens, but uncertainty over visual identification for some species led to a rapid adoption of molecular diagnostics such as PCR in the early 2000s onwards [86] (Fig. 2). Similarly, the use of culture-based methods or incubation on live host leaves (for obligate pathogens like rusts and powdery mildews) also reduced, as molecular diagnostic methods became popular. Whereas some samplers, such as cyclones, collect the sample directly into tubes, many established samplers require an initial processing step such as cutting a tape from a Hirst-type 7-day spore trap (e.g. Burkard or Lanzoni) into a daily section and then lengthways into two sub-sections, each of which can fit into a 2 ml screw-top tube [65, 87]. Similarly, rods used for impaction in rotorod traps [88] are often designed to fit into the same 2 ml screw-top tubes for spore disruption (see below). Spores collected onto surfaces may also be concentrated into a small volume using Electrowetting on Dielectric, which can move a droplet over a surface to produce a highly concentrated sample in a single droplet, which was applied to an immunoassay [89]. Samples collected onto filters can be removed by washing the filter, by dissolving it completely in a suitable solvent [90] or by passing foam through the reverse side of the filter, which removes most spores into the foam on the collected side, and as the foam collapses down into a small volume of liquid, the sample can be effectively removed into a relatively small collection tube as a post-capture process. As many spores have a sticky or adhesive surface [91], it is important to check that all spores are removed from a filter or impacted surface to avoid an additional bias that could occur if sticky spores were lost from downstream processing. Furthermore, some hydrophobic spores and those with an electrostatic charge have been shown to be lost either at the top of discarded supernatant or by binding to plasticware during processes such as centrifuging [92].

For all DNA-based diagnostic methods to be applied to air-particulate samples, i.e. PCR, qPCR, ddPCR, DNA sequencing, Loop-mediated Isothermal Amplification (LAMP), recombinase polymerase assays (RPAs) and other isothermal methods, it is usually necessary to release DNA from within spores and other biological particles. Williams *et al*. [86] tested the detection of *Penicillium roqueforti* conidia by PCR as probably the first study to test integrating PCR with air sampling and found that while it was possible to detect >1,000 spores per reaction directly without disrupting them, in the presence of other airborne spores, it was only possible to detect them by adding steps to disrupt the spores and extract the DNA, which enabled detection of ten spores. The standard approach with an environmental air particulate sample has been not only to disrupt the spores but also to purify the DNA. Disruption can be achieved by physically breaking the spores by shaking them using a device such as a FastPrep^®^, grinder or homogenizer, in a buffer with fine glass, ceramic or zirconia beads, acid-washed sand or metal beads [65, 93–95], or disruption with enzymes and detergents [90, 96] or, in some cases, simply heating to 100 °C [18]. For some targets, particularly bacteria, sonication has been shown to work well [97]. However, some methods do not release the DNA from all spores. Heating above 65 °C, for example, bursts spores of *Blumeria graminis*, but most other spores remain intact. Similarly, sonication has been used on fungal spores, but it is important for researchers to check that this approach is effective particularly on tough fungal spores. Whichever method is selected, it is advised to check a subsample under a microscope to verify that all spores have been disrupted to avoid an additional sampling bias in the DNA extraction step. Particularly tough spores include *Alternaria* and most rusts due to thick spore coats and many very small spores because smaller spores are able to withstand deformation forces.

Spore disruption to make a simple crude suspension of released DNA and other cell contents can be sufficient to produce material suitable for use directly in LAMP, RPA and other isothermal assays, which is why such isothermal methods have been adopted for in-field or automated tests. However, inhibition of isothermal assays can still occur, and so at least a simple dilution or filtration step is often advised, and other improvements to make an assay more robust include the addition of BSA, adjusting the concentration of MgSO_4_ and the addition of betaine [18]. For all types of PCR and sequencing, a relatively pure extract of DNA is necessary, which is why these approaches are normally used in a clean molecular biology lab, which often adds a delay for the sample to be transported from the field. Optimization of processes is often needed to avoid loss or denaturation of DNA from some species that was free or released at early stages of extraction, while ensuring sufficient release of DNA from the toughest spores [96]. The same study [96] also used blanks or controls, as clean filters or collection surfaces, mounted on the air sampler, almost immediately replaced without sampling and returned to the lab for processing to assess potential contamination during production, transportation and storage. Other samples generated in the lab can be used to test only for contamination occurring during DNA extraction and a standard routine of using a non-template control tests for contamination of amplification reagents. Although no contamination was found [96], other studies such as [95] did find small traces of bacteria and fungi but concluded that the clean room the devices were tested in was not completely clean, rather than that the air samplers had been contaminated ahead of use. The issue of contamination may be very important for low-biomass studies (clean rooms, high altitude sampling, Arctic sampling, etc.) and in any sampling programme, whether for surveillance of a specific pathogen or for biodiversity studies, it is important not to have the sampling materials or downstream reagents contaminated with anything that could influence the result. Issues of human DNA contamination caused by skin cells of the operators or researchers can be discounted in most DNA-based studies using bioinformatics methods, particularly for studies focussed on fungal spores, where for many common fungi-specific barcoding methods, human DNA would not amplify.

Some DNA sequencing methods (discussed later), such as multiplexed Illumina sequencing, are prone to a small amount of index misassignment or index hopping, where sequencing reads receive the wrong sample index due to contamination of free adapters or unligated primers during library preparation, which results in the output being assigned to the wrong sample. This error can be reduced by using dual indices, where two unique DNA barcodes, called i5 and i7 indexes, are attached to opposite ends of each library fragment, which reduces index misassignment, as reads with unexpected i5–i7 combinations can be removed during demultiplexing. Negative controls and technical replicates also help to identify possible sample mislabelling or contamination. Disruption of spores need not be necessary for application of immunological or aptamer assays if the antigen being detected is on the surface coat of the spore [98]. However, in some cases, disruption can increase sensitivity of the assay if the antigen being detected is an internal component of the spore or cell rather than a component of the surface coat.

### Qualitative *versus* quantitative methods and dealing with inhibition

PCR (i.e. end-point PCR as opposed to quantitative PCR), LAMP and other isothermal methods are qualitative assays, which indicate simply the presence of the target organism or gene above a known detection limit. It is possible to get some quantification from each of these methods by testing a dilution series of the sample in separate tests. Clearly, this will increase costs but can be necessary where a quantitative method is not available. Essentially, a series of two to four dilutions are tested with the same qualitative assay. If only the most concentrated (i.e. neat extract) gives a positive result but none of the dilutions, it can be deduced that the amount of target is low, whereas if a 1/1,000^th^ dilution still tests positive, it can be assumed that the target has very high abundance. Dilutions not only indicate the order of magnitude of presence of the target but can also be a way to test for inhibition; if diluted samples test positive, but the concentrated sample is negative, it indicates that inhibitors were present at sufficient concentration to inhibit the reaction in the concentrated extract.

Quantitative PCR became an established lab standard for quantification of plant pathogens by 2010 [99]. This method can also be inhibited, particularly once there is a reasonably large biomass of air particulates in the sample. McDevitt *et al*. [100] found that samples weighing as little as 50 µg contain sufficient material to cause inhibition. Additional DNA clean-up steps can be used separately or are integrated into DNA extraction kits, e.g. MoBio kits [101] or the addition of BSA and polyvinylpyrrolidone (which is often in commercial clean-up kits) [40] can be used on samples likely to be at risk of inhibition (typically >50 µg and/or heavily pigmented). A further check can be to spike the sample with a small amount of DNA of a completely different organism to the one being quantified and unlikely to be naturally in the sample, e.g. squid DNA added as a control to an air particulate sample, and a separate qPCR is then run for that exogenous control [40]. If that test is negative, it indicates that inhibitors are still present. Alternatively, a small known amount of the target DNA can be spiked into a technical replicate and the usual qPCR run for that target. The technical replicate with the added DNA should give a result indicating at least the amount of DNA that was added, otherwise indicating inhibition and the need to dilute the sample or run an additional clean-up step.

Immunological systems and biosensors can also be quantitative. In the case of immunological tests, either an ELISA can be used, as reported by Wakeham and Kennedy [102], who were able to quantify airborne spores of *Mycosphaerella brassicicola* in a Brassica crop, or alternatively, the strength of the test line generated in a positive lateral flow device or rapid antigen test can be scanned to determine the relative quantity of the target antigen up to a certain limit for that assay. Various biosensors, which usually detect a binding event of aptamers, primers or antibodies, or an electrochemical reaction to the presence of a metabolite, have been developed [103].

### Detection of genetic traits, DNA sequencing and the aerobiome

In addition to detection of certain species by diagnostic tests, one advantage of DNA-based methods such as PCR is the ability to design primers to detect known genetic traits such as fungicide resistance [20, 21], mating type [104] and races or pathotypes [22, 87, 105]. Of course, deposition of spores onto leaves of different host plant varieties or treated with different fungicides is also possible but can take longer to obtain results due to the incubation time needed for symptom development [78].

Rather than use race-specific PCR assays, DNA sequencing can also identify a race of a pathogen when a single pustule on a leaf is sampled and assuming there is an existing database of different races [106]. Even new genetic traits can be identified, and their function can be predicted using tools such as PHI-base [107]. However, it is much harder to deduce the same level of information from a mixed sample of spores in a spore trap sample due to low biomass and the sample representing a mixture of DNA of different races and species in a tube. It could be possible to use a proximity ligation step to link DNA within the same cell so that a combination of mutations within individual cells only could be determined. Alternatively, individual spores need to be separated, their genome amplified and then sequenced. Further work is in progress to develop rapid sequence-based methods for spore trap samples, e.g. for myrtle rust detection in Australia, a multiplexed Nanopore sequence-based diagnostic assay was developed with automated bioinformatic reporting of specific strains [108, 109].

More frequently, DNA sequencing applied to air-particulate samples has been used to investigate species diversity and relative abundance, otherwise known as the aerobiome [27, 45, 69, 70, 110–112]. Metabarcoding of genetic regions such as ITS (Internal Transcribed Spacer) has been used for fungi, but some fungi do not amplify depending on the primers used [65], and for some barcoding genes such as TEF (Translation Elongation Factor), there is currently (in 2026) a very limited database, so spore trap samples often return a high percentage of ‘unknowns’ or return a genus that is actually only a partial relative, if the level of precision is set very low on the software being used.

In terms of the number of reads as an indication of relative abundance, it should be noted that some barcoding genes may have multiple copies in some species compared to others, or there may be several nuclei per spore, which could skew the apparent relative abundance from the true abundance of spores of a certain species. Therefore, the number of reads can be an indication of spore concentration but needs further refinement such as comparisons with qPCR data for each species of interest, which explains why read counts were only weakly correlated with spore concentrations measured by qPCR [45].

Nanopore sequencing has advantages of providing long reads, and due to sequencing DNA in both directions, the method has improved to have few errors. Additionally, it has been shown that the nanopore raw data allows an indication, using an AI-based model, of whether an organism is viable or not due to subtle changes in the structure of DNA after cell death [113]. Certainly, it would help such studies if samples were either frozen or processed immediately after sampling ended for such a system to avoid mortality of particles while waiting to be processed.

Airborne DNA of microbes was recovered, shotgun-sequenced and taxonomically classified, including down to the species level, to show change in abundance at field-level scale to identify the presence of agriculturally significant pathogens over time [27]. In addition to the detection of important plant pathogens, the presence of known fungal antagonists of certain plant pathogens was also detected [45]. Sequencing methods have the potential to offer vastly improved monitoring of the aerobiome, providing information not only on the abundance of pathogens but also on beneficial fungi such as antagonists or mycoparasites of pathogens. Further work is required to understand the relationship of airborne communities to their in-field impact on disease development. Indeed, the rapidly evolving space of eDNA research and its applications was recently reported [114] following a workshop of participants across a broad spectrum of use cases in Australia. A primary challenge was improving the understanding of airborne eDNA ecology, harmonizing sampling methodology (e.g. devices, materials, sampling density and duration), identifying and mitigating sources of error and fostering early, sustained stakeholder collaboration. Overcoming these challenges would see airborne eDNA analysis recognized as a transformative tool for biodiversity, biosecurity and conservation monitoring on a global scale.

### Concluding discussion

There is clearly a wealth of information in the scientific literature that has been amassed in the past 70 years since Hirst developed a spore trap specifically to study associations between airborne spores, weather and plant disease [4]. This review has highlighted some key aspects and context within the literature to guide emerging researchers in this area of science, as well as key aspects for consideration both for practical plant health or biodiversity surveillance and aerobiological research. These considerations apply to researchers and applied scientists, investigating fungal spores in the air, whether by using DNA sequencing or more classical methods, including sampler choice, position, frequency of collection and density of sampling positions; post-capture handling and, particularly, issues of DNA extraction, handling inhibitors and having adequate checks and controls or reference materials for image-based methods; and an understanding of atmospheric physics, spore dispersal and viability and fungal biology.

It is important for any study involving the measurement of airborne biological particles to report essential minimum information (Table 1) – sampler type (and adhesive used for impaction samplers), height of sampler above ground, sampler air flow rate, surrounding vegetation and analysis method (and fine methodology such as DNA extraction, checks for inhibitors and calibration with standards). It is also important to consider the integrity and representational context of a sample and limitations of the diagnostic assay applied to it. Analysis using traditional microscopy has reduced in use due to time constraints and uncertainty, but where it is used, including by image analysis or machine vision, the minimum requirements set out for pollen monitoring should be considered [115]. In all cases, the air itself should never be considered as the source of spores.

**Table 1.** Minimum information suggested to be included in research papers on aerobiological or bioaerosol studies

Primary information	Secondary information	Note
**Sampler type and make**	Collection surface and adhesive (for impactors). Rod dimensions, rotation speed and radius of rotorods. Filter size and type (for filters). Collection liquid (for impingers and wet cyclones). Collection tube size and type (for cyclones and virtual impactors).	A detailed description or reference should be given for custom-fabricated devices. Optional extra information includes power supply (e.g. mains, solar or battery)
**Height of sampler above ground**	Add height above plant canopy for uses in vegetation	Add a note of surrounding vegetation type or terrain
**Volume of air sampled**	Method and frequency of checks for sample volume	Air flow rate or rotation speed of rotorods
**Sample analysis method**	DNA or antigen release and extraction method (for DNA and immunological methods) Microscopy methods (including aided by image analysis or machine learning) should follow minimum requirements set out for pollen monitoring [115].	Add methods for checks for inhibition (for DNA and immunological methods), methods of calibration or standards used (including reference image sources)

Thresholds for action when sampling outdoor air are extremely difficult to set due to spore concentrations varying by orders of magnitude with height and proximity to the source, which is why a compromise is often reached in sampling position to elevate the sampler to obtain a larger representative area while maintaining an ability to detect relatively close sources, and some preliminary or previous studies are usually helpful to identify typical concentrations associated with an important spore release event. It is helpful if studies report spore concentrations in air (e.g. spores per m^3^) to allow comparison between sites, and this can usually be estimated from amounts of DNA or numbers of reads, although the translation to spore concentrations varies according to the assay and pathogen.

Methods exist to assess spore viability, whether by culturing, triggering germination [59, 116] for imaging or an immunological detection of mycelial surface antigens, detecting RNA (few studies have done so without a culturing step) or use of vital stains with microscopy. Culturing itself is problematic due to many species not being able to be grown on artificial media and slower-growing species being missed due to overgrowth by fast-growing species [44]. However, usually the detection of large concentrations of a target species is sufficient to indicate that at least some of those spores will still be viable. Although quantitative analysis is best, qualitative detection methods that can be completed quickly, and even automated, are often sufficient to aid crop protection decisions. This can be particularly effective if the sensitivity of the assay is designed to detect a significant concentration of spores above a negligible background concentration typically present before a monocyclic pathogen starts a seasonal release of spores locally or before a polycyclic pathogen has built up to a significant local population size. Automation of systems to detect damaging plant pathogens is likely to continue with DNA-based assays, immunological assays, biosensors or optical systems, but uptake by farmers and agronomists to assist crop protection decisions will only occur if such systems are easy to use, relatively cheap and very reliable. In addition, for high-value crops, such a system needs to be cheap enough to allow multiple sampling positions within a crop to reduce the chance of an initial small source of spores being missed.

Future research on biodiversity using air sampling will probably use AI or automated processes increasingly to analyse either DNA sequence information or image- and spectroscopy-based data. However, for both approaches, databases of sequences or optical data will need to be improved in order to increase accuracy, particularly as DNA sequencing methods often detect genera that are not noticed visually [117]. The choice of bar-coding genetic regions to use is also not currently standardized. While optical methods can already give same-day or even real-time data of ‘spore type’, the speed of DNA sequencing methods is currently much slower but is likely to improve, which AI will almost certainly contribute to. However, it is also important to note that although airborne biodiversity can indicate the status of local ecosystems, care in interpretation is needed because some of the sample is likely to have come from a long way away. It seems that some standard is needed to consider an organism to be of local origin only if it occurs in air on multiple occasions within a season and when the wind is blowing in at least half of the possible compass directions.

Given the complexity of factors that influence aerobiological systems, it is not surprising that no standardized method of air sampling has been established. This is because of differences in the suitability of different samplers depending on the purpose of sampling, size of target particles, issues of sample recovery and analysis, cost, sampling height or platform and number of sampling positions required. One aim of this review has been to highlight factors for consideration in designing a sampling programme, whether for academic or practical purposes.

## Abbreviations

LAMP: Loop-mediated Isothermal Amplification
MTIST: Microtitre Immuno Spore Trap
RPAs: recombinase polymerase assays
